# Clinicopathological Features of Dacryolithiasis in Japanese Patients: Frequent Association with Infection in Aged Patients

**DOI:** 10.1155/2013/406153

**Published:** 2013-09-02

**Authors:** Masabumi Kubo, Tomoki Sakuraba, Ryuichi Wada

**Affiliations:** ^1^Fukiage Eye Clinic, 2-10 Fukiage, Hachinohe 031-0003, Japan; ^2^Department of Ophthalmology, Aomori Prefectural Central Hospital, 2-1-1 Higashi Tsukurimichi, Aomori 030-8553, Japan; ^3^Department of Pathology and Molecular Medicine, Hirosaki University Graduate School of Medicine, 5 Zaifu-cho, Hirosaki 036-8562, Japan

## Abstract

*Purpose*. The purpose of this study is to elucidate the clinicopathological features of dacryolithiasis and prevalence of associated infection in Japanese patients. *Materials and Methods*. Out of 13,471 outclinic patients from 2006 to 2011, 268 patients were scheduled to be performed dacryocystorhinostomy (DCR) due to dacryocystitis with nasolacrimal duct obstruction. Actually 266 patients underwent dacryocystorhinostomy (DCR) and two patients were cured by only ophthalmic examination. Dacryoliths were found in 17 cases (6.3%). Among 17 cases of dacryolithiasis, three patients were male, and 14 were female. The age of the patients ranged from 32 to 82 (mean 67) years, and 13 cases (76%) were more than 65 years of age. Pathological examination disclosed the infectious agents in 9 cases (53%), and all patients with infection were more than 65 years of age. Special stains revealed colonies of fungus, suspicious of *Aspergillus*, in 6 cases and gram-positive rods, and suspicious of *Actinomyces*, in 3 cases. *Conclusions*. The current study showed the frequent association of infection with dacryolithiasis in aged Japanese patients. This should be taken into the consideration for the treatment of dacryolithiasis.

## 1. Introduction

Dacryolithiasis is a frequent disease of the lacrimal system, and middle-aged women are commonly affected [1–8]. The pathogenesis of dacryolithiasis is not well understood. The association of infection with dacryolithiasis was reported in the literature [1–8]; however, the prevalence of infection associated with dacryolithiasis in Japanese patients is not known to date. The aim of the current study is to elucidate the clinicopathological features of dacryolithiasis and the prevalence of associated infection in Japanese patients who underwent dacryocystorhinostomy (DCR).

## 2. Materials and Methods

From the archival files of 13,471 patients of the Fukiage Eye Clinic from 2006 to 2011, 268 clinical records of dacryocystitis with nasolacrimal duct obstruction were retrieved, and dacryolithiasis was noticed in 17 cases (Table 1). There was no immune compromise patient in these 17 cases. We did not perform dacryocsytogram and lacrimal endoscopy before DCR; therefore, we have no precise information with nasolacrimal dust obstruction. Cefcapene pivoxil hydrochloride hydrate was continued for four days after the procedure, along with topical antibiotic eye drop and steroid eye drop four times a day for three months. The clinical records and pathological reports of these patients were reviewed. Informed consent was obtained from the patients.

Dacryoliths and biopsy specimens obtained from 17 cases of DCR were fixed in 10% buffered formalin and subjected to pathological examinations. Sections were stained with hematoxylin and eosin, periodic acid-Schiff, Gram method, and Grocott staining.

## 3. Results

**Figure 1:**
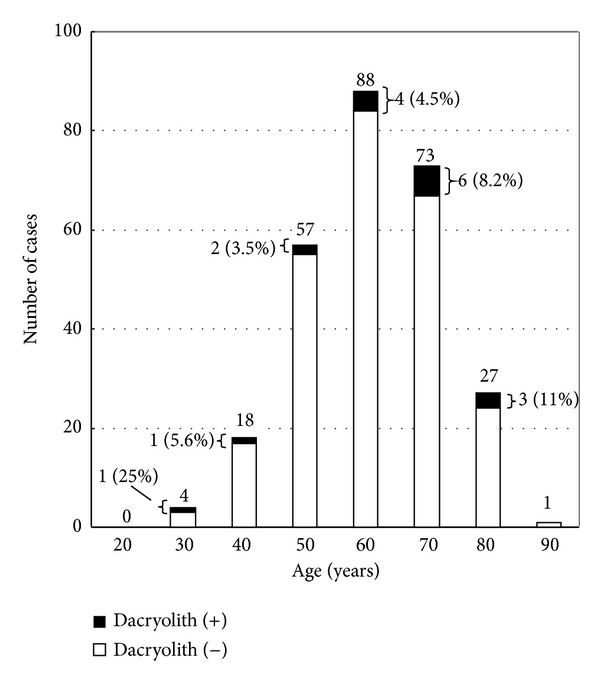
The age distribution of the patients treated by dacryocystorhinostomy. The closed box indicates the patients with dacryoliths.

Total 268 patients were scheduled to undergo DCR due to dacryocystitis with nasolacrimal duct obstruction (Figure 1). Actually 266 patients underwent DCR. In two patients, dacryoliths were removed by lacrimal endoscopy and DCR was not performed.

Most of the patients were aged at the sixth and seventh decades (Figure 1). Out of 268 cases dacryoliths were found in 17 cases (6.3%) (Figure 1). The prevalence of dacryolithiasis increased over years from 3.5% to 11% in the patients of age from 50 to 80 (Figure 1).

**Figure 2:**
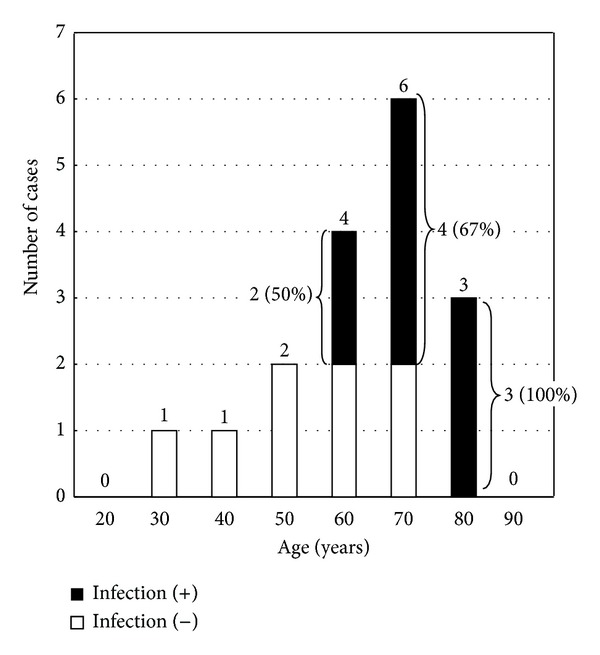
The age distribution of dacryolithiasis. Closed box indicates the case associated with infection.

The clinicopathological features of cases of dacryolithiasis were summarized in Table 1. The age of the patients of dacryolithiasis ranged from 32 to 82 (mean 67) years and 13 cases (76%) were more than 65 years. Three patients (18%) were male, and 14 patients (82%) were female. All patients presented with persistent epiphora, pus discharge, and swelling of the lacrimal sac. No patient had a history of intermittent epiphora. Two patients had suffered from the disease more than 10 years. Three patients had past history of silicone intubation, and diabetes was noted in one case. Dacryoliths were found in lacrimal sac in 16 cases and both in lacrimal sac and nasolacrimal duct in one case. In 8 out of 17 patients (47%), the biopsy specimens were mucinous materials and granulations tissues, and no infectious agents were identified (Table 1). Microorganisms were identified in dacryoliths of 9 patients (53%). All cases with infection were more than 65 years of age, whereas the infectious agent was not identified in 4 patients of age less than 65 years (Figure 2). The prevalence of infection showed gradual increase over the age, and they were 50%, 67%, and 100% in the sixth, seventh and eighth decades, respectively.

**Figure 3:**
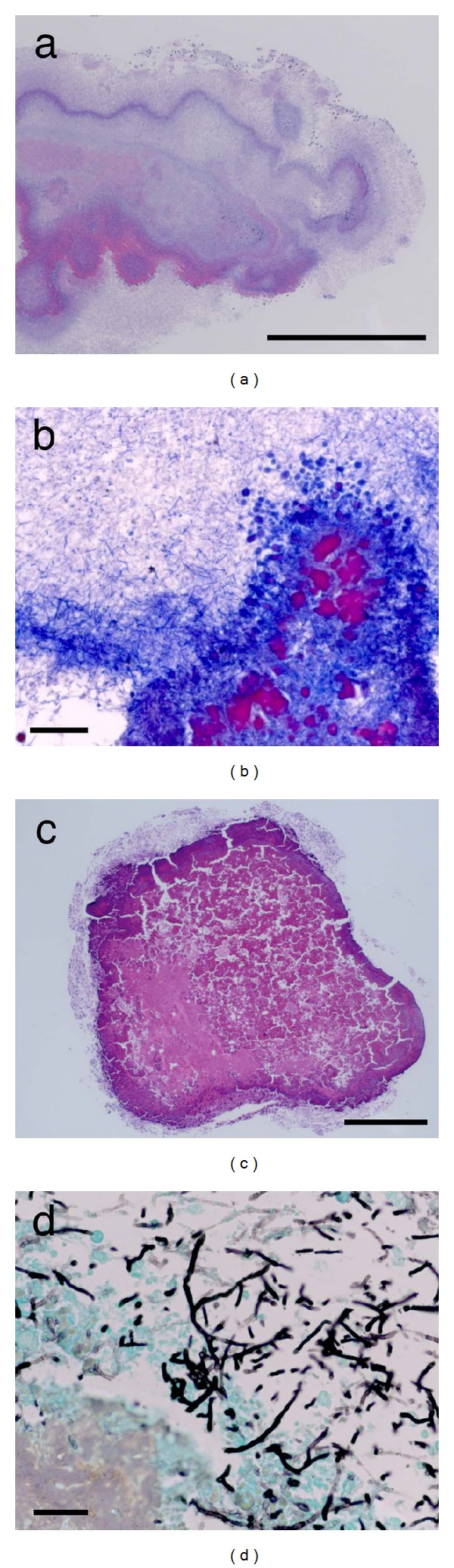
Pathology of dacryoliths. (a) Colony of gram-positive rods in case 11. H and E staining; bar = 0.5 mm. (b) Gram staining; bar = 20 *μ*m. (c) Colony of fungus in case 15. H and E staining; bar = 0.5 mm. (d) Gomori's methenamine silver staining; bar = 20 *μ*m.

Colonies of gram-positive rods were found in 3 cases (Figures 3(a) and 3(b)). The morphological features of the colony were consistent with sulfur granule of *Actinomyces* species (Table 1). Fungi were demonstrated in 6 cases (Table 1). Fungal hyphae with occasional Y-shaped branch and septae, which was consistent with *Aspergillus*, were demonstrated by Grocott staining (Figures 3(c) and 3(d)).

## 4. Discussion

The current study demonstrated that the aged people older than 65 years is affected by dacryolithiasis and that the disease is frequently associated with infection of microorganisms. The current study is the first report that showed the prevalence of infection associated with dacryolithiasis in Japan. To estimate the precise prevalence of infection, the dacryoliths were pathologically examined and infectious agents were identified with special stains.

The prevalence of dacryoliths in the patients who underwent DCR and lacrimal endoscopy was 6.3% in the current study. The prevalence varied from 6.7% to 17.0% in the previous studies (Table 2). The prevalence observed in the current case was slightly low, but the value was comparable with those in the previous reports (Table 2). It should be noted that the prevalence of dacryolithiasis showed gradual increase over years more than 50 years (Figure 1). The mean age of the patients in the current study was higher than those of the previous studies at least by 8 years (Table 2). This may reflect the increase in the aged people in Hachinohe city, where the clinic is located, and the current study may highlight the clinical features of dacryolithiasis of aged people. Although the previous studies suggested the frequent occurrence of dacryolithiasis in middle-aged people [1, 3–8], it should be remembered that dacryolithiasis affects not only middle-aged women but also aged people over 65 years in Japan.

The pathogenesis of formation of dacryolith is not well known [1–8]. Female predominance in the patients of dacryolithiasis was reported in the previous studies [1–8], and this is also the case in the current study. It was suggested that the powder of cosmetics can form a core of dacryolith and may also induce the growth of microorganism [7]. Congenital dacryocystitis is a very rare disease and may cause dacryolithiasis in young patients [5]. In the current study, such young patient was not found in current study. It is controversial whether smoking is a risk factor [6]. Three patients had a history of canalicular intubation of silicone tube. The insertion of foreign body in the lacrimal system may be a risk factor for development of dacryolith, especially in aged people. 

Previous studies revealed the association of infection dacryolithiasis, and the prevalence of infection varied largely from 0% to 87% (Table 2). In the current study, the prevalence of associated infection was 53%, and the value was comparable with those of the previous studies. It is of note that the prevalence increased over years in the patients more than 65 years of age (Figure 2). 

When dacryocystitis is accompanied with dacryolith and nasolacrimal duct obstruction, surgical treatment is necessary. Treatment antibiotics alone may not be effective. Since fungus and* Actinomyces* are frequent pathogen, this should be taken into the consideration for the selection of antibiotics. As shown in the current study, the prevalence of infection is high in aged patients with dacryolithiasis. Infections agent should be carefully examined in the dacryolith for the postoperative treatment and prevention of recurrence.

The number of patients in the current study is limited. Further investigation is required for a better understanding of the pathogenesis of dacryoliths and treatments for nasolacrimal obstruction.

## Figures and Tables

**Table 1:** Clinicopathological features of cases of dacryolithiasis.

Case no.	Age/sex	Symptoms	Duration	Background	No. of acute dacryocystitis	Location	Pathology/identified microorganisms
1	32/M	Epiphora, pus	6 years	—	3	LS/NLD	Mucinous material
2	47/M	Epiphora, pus	1 year	lacrimal endoscopy	1	LS	Mucinous and degenerated tissue
3	53/F	Epiphora, pus	6 months	—	0	LS	Granulation tissue
4	54/F	Epiphora, pus	3 years	lacrimal endoscopy	0	LS	Proteus material
5	65/M	Epiphora, pus	3 years	—	0	LS	*Aspergillus*
6	65/F	Epiphora, pus	>10 years	—	0	LS	Degenerated tissue
7	68/F	Epiphora, pus	1 year	Silicone intubation	0	LS	Granulation tissue
8	68/F	Epiphora, pus	2 years	—	0	LS	*Aspergillus *
9	71/F	Epiphora, pus	3 years	Silicone intubation	0	LS	*Aspergillus *
10	71/F	Epiphora, pus	3 years	—	1	LS	*Aspergillus *
11	72/F	Epiphora, pus	2 years	DM	0	LS	Degenerated tissue
12	79/F	Epiphora, pus	3 years	—	0	LS	Granulation tissue
13	79/F	Epiphora, pus	1 year	—	0	LS	*Actinomyces *
14	79/F	Epiphora, pus	1 year	—	0	LS	*Actinomyces *
15	80/F	Epiphora, pus	2 years	—	1	LS	*Actinomyces *
16	82/F	Epiphora, pus	>10 years	Silicone intubation	0	LS	*Aspergillus *
17	82/F	Epiphora, pus	1 year		0	LS	*Aspergillus *

M: male, F: female, DM: diabetes mellitus, LS: lacrimal sac, NLD: nasolacrimal duct.

**Table 2:** Summary of clinicopathological features of dacryolithiasis reported in the literature.

Author	Year	DCR	Dacryolithiasis (%)	Mean age (years)	Male : female	Identified microorganisms			
						Infection (%)	Fungus (%)	Actinomyces (%)	Others (%)
Jones [2]	1965	180	25 (14)	<50, 22 cases >50, 3 cases	10 : 15	0 (0)	0 (0)	0 (0)	—
Berlin et al. [3]	1980	70	11 (15.7)	42	1 : 10	6 (55)	6 (100)	0 (0)	—
Willkins and Pressly [4]	1980	94	16 (17.0)	<50, 7 cases >50, 5 cases	2 : 10	0 (0)	0 (0)	0 (0)	—
Hawes [5]	1988	107	15 (14.0)	45	4 : 11	0 (0)	0 (0)	0 (0)	—
Yazici et al. [6]	2001	163	12 (7.4)	59	8 : 4	2 (17)	2 (100)	0 (0)	—
Marthin et al. [7]	2005	—	62 (—)	59	16 : 46	44 (87)*	12 (22)	32 (59)	19 (35)
Repp et al. [8]	2009	327	22 (6.7)	51	9 : 13	5 (23)	2 (40)	3 (60)	—
Current stydy	2013	268	17 (6.3)	67	3 : 14	9 (53)	6 (67)	3 (33)	—

*Six cases were infected with more than one type of microorganism.
